# Feasibility of Older Adult Remote Self‐Installation of Passive Sensor Assessment Technologies

**DOI:** 10.1002/alz.092868

**Published:** 2025-01-09

**Authors:** Sarah Gothard, Nathaniel Rodrigues, Stephanie Irish, Jeffrey A Kaye, Zachary T Beattie, Allison Lindauer

**Affiliations:** ^1^ Center for Health & Healing Building, 3303 S Bond Ave, 1 8th Floor, Portland, OR USA; ^2^ NIA‐Layton Aging & Alzheimer’s Disease Research Center, Portland, OR USA; ^3^ Oregon Health & Science University, Portland, OR USA; ^4^ Oregon Center for Aging & Technology (ORCATECH), Portland, OR USA; ^5^ Oregon Health and Science University, Portland, OR USA; ^6^ Oregon Health Science University, Portland, OR USA; ^7^ NIA‐Layton Aging & Alzheimer’s Disease Center, Portland, OR USA

## Abstract

**Background:**

Conducting research remotely in aging and Alzheimer’s disease related (ADRD) populations using multiple passive sensing technologies (e.g., activity watches, electronic pillboxes, bed‐mats, wall‐mounted sensors) provides opportunities for greater inclusiveness and more ecologically valid data capture. A challenge to this approach has been the need to enter homes to deploy and/or maintain devices or technologies. To overcome this limitation, we developed and tested a novel protocol for entirely remote self‐deployment of technologies.

**Method:**

Research participants in 14 households (age = 70.8±7.2 years; 61.5% female; education = 16.5±2 years; telephone MoCA = 21±1.6, days of follow‐up = 834.9±604.7) in an aging and dementia study were sent an online accessible Remote Installation Manual to guide independent installation of technologies in their homes. The manual included technology overviews, video installation tutorials, and written step‐by‐step instructions for each device. Eight separate sensing technologies could be deployed in each home (PIR motion sensors, contact door sensors, activity watches, wireless body composition scale, bed mats, driving sensors, and a data hub aggregating computer). Participants could request installation support, if necessary. The success of self‐deployment was compared to 22 study homes, which were installed in‐person by study field technicians.

**Result:**

Four of 14 participants deployed the technologies entirely using only supplied instructions: 10/14 participants required assistance with at least one aspect of installation; all issues were resolved through remote contact. Data recovery and maintenance issues between the participant‐installed homes and the 22 technician‐installed homes were not different. The median percentage of days with technical issues were similar (volunteer = 2.3%; technician = 1.4%). The percentage of days of captured data for watches, bed‐mats, and electronic pillbox use were similar or better for volunteer‐installed homes (watch: volunteer = 72.9%, technician = 70.6%; bed‐mat: volunteer = 72.0%, technician = 67.2%; pillbox: volunteer = 72.0%, technician = 68.5%).

**Conclusion:**

Older adults are readily able to deploy remote assessment technologies without study personnel needing to travel to or enter a residence. Data generation and technical issues related to the research technologies are comparable between study participant self‐deployment and technician‐deployment, demonstrating feasibility and effectiveness of remote older adult self‐deployment of study technologies. The ability to deploy digital technologies for continuous, objective and ecologically valid assessments anywhere with internet access may greatly broaden the inclusiveness of ADRD research.

